# (μ-2,3-Dihydroxy­butane-1,4-dithiol­ato)bis­[triphenyl­tin(IV)]

**DOI:** 10.1107/S1600536809055135

**Published:** 2010-01-09

**Authors:** Cuiping Li, Rufen Zhang

**Affiliations:** a College of Chemistry and Chemical Engineering, Liaocheng University, Shandong 252059, People’s Republic of China

## Abstract

In the title compound, [Sn_2_(C_6_H_5_)_6_(C_4_H_8_O_2_S_2_)], the geometry around the Sn atoms is distorted tetra­hedral. The hydr­oxy groups are involved in O—H⋯O hydrogen bonding, which connects mol­ecules into centrosymmetric dimers.

## Related literature

For related structures, see: Basu Baul (2008 ▶); Ma & Zhang (2006 ▶).
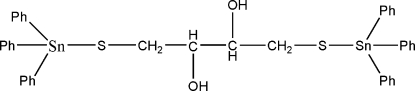


## Experimental

### 

#### Crystal data

[Sn_2_(C_6_H_5_)_6_(C_4_H_8_O_2_S_2_)]
*M* *_r_* = 852.20
Triclinic, 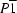

*a* = 10.4806 (4) Å
*b* = 12.3774 (5) Å
*c* = 14.9797 (6) Å
α = 104.656 (1)°
β = 90.470 (1)°
γ = 95.521 (1)°
*V* = 1870.19 (13) Å^3^
*Z* = 2
Mo *K*α radiation
μ = 1.48 mm^−1^
*T* = 293 K
0.25 × 0.22 × 0.21 mm

#### Data collection

Siemens SMART CCD area-detector diffractometer
Absorption correction: multi-scan (*SADABS*; Sheldrick, 1996 ▶) *T*_min_ = 0.709, *T*_max_ = 0.746
21325 measured reflections
6551 independent reflections
5739 reflections with *I* > 2σ(*I*)
*R*_int_ = 0.019

#### Refinement

*R*[*F* ^2^ > 2σ(*F* ^2^)] = 0.023
*wR*(*F* ^2^) = 0.057
*S* = 1.06
6551 reflections
417 parameters
H-atom parameters constrained
Δρ_max_ = 0.29 e Å^−3^
Δρ_min_ = −0.51 e Å^−3^

### 

Data collection: *SMART* (Siemens, 1996 ▶); cell refinement: *SAINT* (Siemens, 1996 ▶); data reduction: *SAINT*; program(s) used to solve structure: *SHELXS97* (Sheldrick, 2008 ▶); program(s) used to refine structure: *SHELXL97* (Sheldrick, 2008 ▶); molecular graphics: *SHELXTL* (Sheldrick, 2008 ▶); software used to prepare material for publication: *SHELXTL*.

## Supplementary Material

Crystal structure: contains datablocks I, global. DOI: 10.1107/S1600536809055135/gk2249sup1.cif

Structure factors: contains datablocks I. DOI: 10.1107/S1600536809055135/gk2249Isup2.hkl

Additional supplementary materials:  crystallographic information; 3D view; checkCIF report

## Figures and Tables

**Table:** 

Sn1—S1	2.4159 (8)
Sn2—S2	2.4086 (8)

**Table:** 

C11—Sn1—S1	108.60 (8)
C17—Sn1—S1	118.70 (7)
C5—Sn1—S1	101.47 (7)
C35—Sn2—S2	107.68 (8)
C29—Sn2—S2	105.19 (8)
C23—Sn2—S2	107.00 (7)

**Table 2:** Hydrogen-bond geometry (Å, °)

*D*—H⋯*A*	*D*—H	H⋯*A*	*D*⋯*A*	*D*—H⋯*A*
O1—H1⋯O2^i^	0.82	1.95	2.745 (3)	163

Symmetry code: (i) 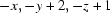.

**Fig. 1.**
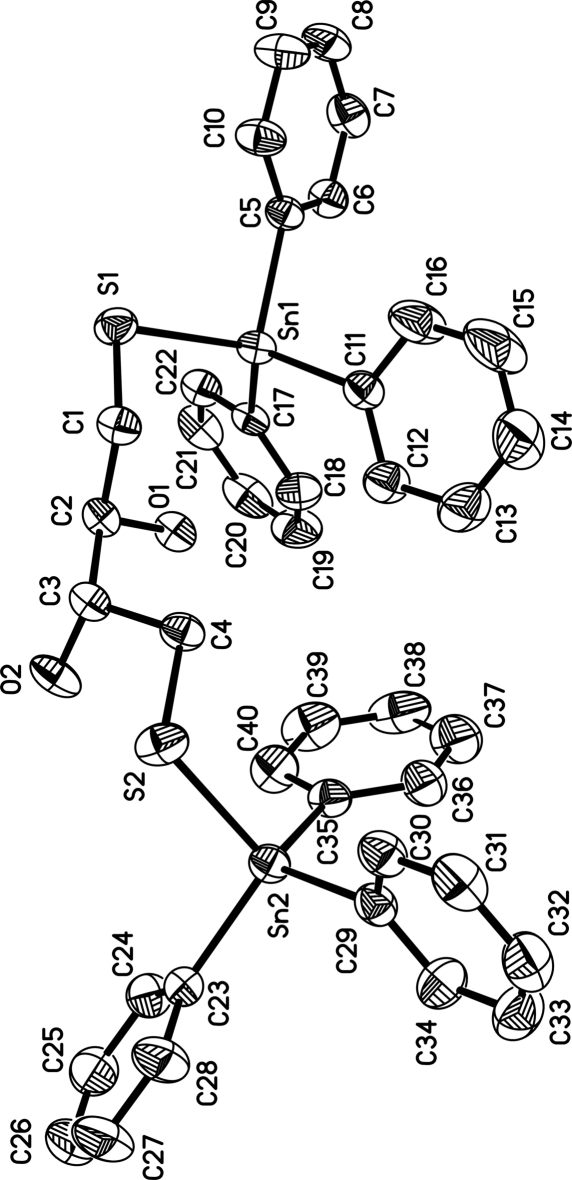
The molecular structure of the compound, showing 30% probability displacement ellipsoids. H atoms have been omitted for clarity.

**Fig. 2.**
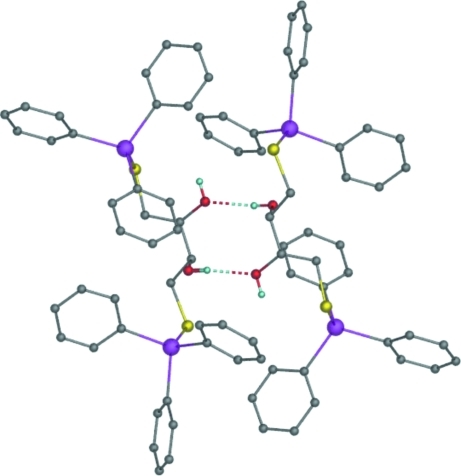
The dimer structure of the compound via O—H···O hydrogen-bonding. Hydrogen bonds are shown with dashed lines.

## supplementary crystallographic information

### Comment

Since some triphenyltin(IV) compounds have been found to exhibit antimicrobial activity, varieties of triorganotin(IV) compounds have been synthesized and studied in the context of their antimicrobial potential (Basu Baul, 2008). 1,4-dithioerythritol is a protective agent for preventing oxidation of thiol groups and a reagent for the reduction of disulfide groups in proteins. Our interest has been focused on studying the reaction under a mild condition and hoping to obtain a new organotin complex with potential biological activities. Here, we have synthesized the title compound and present its crystal structure. The title compound, which is shown in Fig.1 forms a dimer structure by O—H···O hydrogen bonding. The ligand is coordinated to Sn atoms by the sulfur atoms. The Sn—S bond distances in the compound (Sn(1)—S(1) = 2.416 (7) Å; Sn(2)—S(2) = 2.4087 (8) Å) are comparable to those found in related organotin complexes (Ma *et al.*, 2006). The Sn atom assumes a distorted tetrahedron geometry defined by three carbon atoms of the three phenyl groups and one sulfur atom of the dithioerythriol fragment.

### Experimental

The reaction was carried out under nitrogen atmosphere. 1,4-Dithioerythritol (1 mmol) and sodium ethoxide (2 mmol) were added to a stirred solution of benzene (30 ml) in a Schlenk flask and stirred for 0.5 h. Triphenyltin chloride (2 mmol) was then added to the reactor and the reaction mixture was stirred for 12 h at room temperature. The resulting clear solution was evaporated under vacuum. The product was crystallized from ether to yield colourless blocks of compound (yield 81%;. m.p.355 K). Anal. Calcd (%) for C_40_H_38_O_2_S_2_Sn_2_ (Mr = 852.20): C, 56.37; H, 4.49; Found (%): C, 56.01; H, 4.05.

### Refinement

The H atoms were positioned geometrically, with methylene C—H distances of 0.97 Å, methine C—H distances of 0.98 Å, hydroxy O—H distances of 0.82 Å and aromatic C—H distances of 0.93 Å, and refined as riding on their parent atoms with *U*_iso_(H) = 1.2 *U*_eq_(C) or 1.5 *U*_eq_(O).

### Crystal data

**Table:**

[Sn_2_(C_6_H_5_)_6_(C_4_H_8_O_2_S_2_)]	*Z* = 2
*M**_r_* = 852.20	*F*(000) = 852
Triclinic, *P*1	*D*_x_ = 1.513 Mg m^−^^3^
*a* = 10.4806 (4) Å	Mo *K*α radiation, λ = 0.71073 Å
*b* = 12.3774 (5) Å	Cell parameters from 6651 reflections
*c* = 14.9797 (6) Å	θ = 2.4–28.1°
α = 104.656 (1)°	µ = 1.48 mm^−^^1^
β = 90.470 (1)°	*T* = 293 K
γ = 95.521 (1)°	Block, colorless
*V* = 1870.19 (13) Å^3^	0.25 × 0.22 × 0.21 mm

### Data collection

**Table:**

Siemens SMART CCD area-detector diffractometer	6551 independent reflections
Radiation source: fine-focus sealed tube	5739 reflections with *I* > 2σ(*I*)
graphite	*R*_int_ = 0.019
φ and ω scans	θ_max_ = 25.0°, θ_min_ = 1.9°
Absorption correction: multi-scan (*SADABS*; Sheldrick, 1996)	*h* = −10→12
*T*_min_ = 0.709, *T*_max_ = 0.746	*k* = −14→14
21325 measured reflections	*l* = −17→17

### Refinement

**Table:**

Refinement on *F*^2^	Primary atom site location: structure-invariant direct methods
Least-squares matrix: full	Secondary atom site location: difference Fourier map
*R*[*F*^2^ > 2σ(*F*^2^)] = 0.023	Hydrogen site location: inferred from neighbouring sites
*wR*(*F*^2^) = 0.057	H-atom parameters constrained
*S* = 1.06	*w* = 1/[σ^2^(*F*_o_^2^) + (0.0235*P*)^2^ + 0.9814*P*] where *P* = (*F*_o_^2^ + 2*F*_c_^2^)/3
6551 reflections	(Δ/σ)_max_ = 0.001
417 parameters	Δρ_max_ = 0.29 e Å^−^^3^
0 restraints	Δρ_min_ = −0.51 e Å^−^^3^

### Fractional atomic coordinates and isotropic or equivalent isotropic displacement parameters (Å^2^)

**Table:**

	*x*	*y*	*z*	*U*_iso_*/*U*_eq_	
Sn1	0.268290 (16)	0.706634 (14)	0.364645 (12)	0.04754 (6)	
Sn2	0.148861 (17)	1.096900 (14)	0.869657 (12)	0.05017 (6)	
S1	0.32886 (8)	0.88504 (6)	0.33085 (5)	0.06081 (18)	
S2	0.26878 (8)	1.17374 (6)	0.75957 (5)	0.0673 (2)	
O1	0.14670 (17)	0.89633 (14)	0.49766 (13)	0.0532 (4)	
H1	0.0717	0.8868	0.4790	0.080*	
O2	0.08474 (18)	1.11808 (17)	0.59391 (15)	0.0680 (6)	
H2	0.0845	1.1662	0.6429	0.102*	
C1	0.3363 (3)	0.9801 (2)	0.44574 (18)	0.0538 (6)	
H1A	0.3855	0.9494	0.4869	0.065*	
H1B	0.3811	1.0515	0.4433	0.065*	
C2	0.2049 (2)	0.9995 (2)	0.48472 (17)	0.0468 (6)	
H2A	0.1525	1.0222	0.4394	0.056*	
C3	0.2131 (2)	1.0918 (2)	0.57454 (17)	0.0480 (6)	
H3	0.2637	1.1583	0.5647	0.058*	
C4	0.2726 (3)	1.0593 (2)	0.65489 (17)	0.0534 (6)	
H4A	0.3606	1.0441	0.6420	0.064*	
H4B	0.2256	0.9917	0.6639	0.064*	
C5	0.3487 (2)	0.5930 (2)	0.25173 (17)	0.0495 (6)	
C6	0.2951 (3)	0.4833 (2)	0.2183 (2)	0.0592 (7)	
H6	0.2203	0.4588	0.2435	0.071*	
C7	0.3508 (3)	0.4094 (2)	0.1481 (2)	0.0711 (9)	
H7	0.3138	0.3358	0.1267	0.085*	
C8	0.4600 (3)	0.4441 (3)	0.1101 (2)	0.0763 (9)	
H8	0.4977	0.3943	0.0631	0.092*	
C9	0.5134 (3)	0.5517 (3)	0.1412 (2)	0.0813 (10)	
H9	0.5875	0.5757	0.1150	0.098*	
C10	0.4586 (3)	0.6258 (3)	0.2114 (2)	0.0676 (8)	
H10	0.4964	0.6992	0.2319	0.081*	
C11	0.3686 (3)	0.7025 (2)	0.48780 (19)	0.0553 (6)	
C12	0.3202 (3)	0.7338 (3)	0.5750 (2)	0.0703 (8)	
H12	0.2406	0.7616	0.5825	0.084*	
C13	0.3891 (4)	0.7242 (3)	0.6515 (2)	0.0896 (11)	
H13	0.3544	0.7438	0.7096	0.107*	
C14	0.5063 (5)	0.6865 (4)	0.6420 (3)	0.1076 (15)	
H14	0.5525	0.6807	0.6935	0.129*	
C15	0.5558 (4)	0.6574 (5)	0.5573 (3)	0.1245 (19)	
H15	0.6367	0.6318	0.5509	0.149*	
C16	0.4885 (4)	0.6649 (4)	0.4801 (3)	0.0962 (13)	
H16	0.5242	0.6445	0.4224	0.115*	
C17	0.0695 (2)	0.6466 (2)	0.35832 (18)	0.0491 (6)	
C18	0.0085 (3)	0.6228 (3)	0.4331 (2)	0.0695 (8)	
H18	0.0530	0.6374	0.4896	0.083*	
C19	−0.1188 (4)	0.5772 (3)	0.4252 (3)	0.0876 (11)	
H19	−0.1587	0.5605	0.4760	0.105*	
C20	−0.1855 (3)	0.5567 (3)	0.3428 (3)	0.0843 (11)	
H20	−0.2708	0.5264	0.3377	0.101*	
C21	−0.1268 (3)	0.5806 (3)	0.2681 (3)	0.0754 (9)	
H21	−0.1724	0.5676	0.2122	0.090*	
C22	0.0002 (3)	0.6243 (2)	0.2755 (2)	0.0601 (7)	
H22	0.0399	0.6391	0.2240	0.072*	
C23	0.0050 (3)	1.2060 (2)	0.91919 (18)	0.0540 (6)	
C24	−0.1185 (3)	1.1839 (3)	0.8829 (2)	0.0702 (8)	
H24	−0.1409	1.1188	0.8365	0.084*	
C25	−0.2100 (4)	1.2566 (4)	0.9142 (3)	0.0866 (11)	
H25	−0.2929	1.2407	0.8885	0.104*	
C26	−0.1792 (5)	1.3503 (4)	0.9816 (3)	0.0985 (13)	
H26	−0.2411	1.3987	1.0032	0.118*	
C27	−0.0575 (5)	1.3747 (4)	1.0187 (3)	0.1061 (14)	
H27	−0.0364	1.4401	1.0649	0.127*	
C28	0.0348 (4)	1.3026 (3)	0.9878 (2)	0.0831 (10)	
H28	0.1175	1.3197	1.0136	0.100*	
C29	0.2827 (3)	1.1052 (2)	0.97950 (19)	0.0562 (7)	
C30	0.4117 (3)	1.1355 (2)	0.9722 (2)	0.0643 (7)	
H30	0.4409	1.1533	0.9187	0.077*	
C31	0.4979 (4)	1.1397 (3)	1.0439 (3)	0.0791 (10)	
H31	0.5846	1.1604	1.0384	0.095*	
C32	0.4560 (4)	1.1139 (3)	1.1217 (3)	0.0842 (11)	
H32	0.5147	1.1157	1.1691	0.101*	
C33	0.3296 (4)	1.0853 (3)	1.1320 (2)	0.0867 (11)	
H33	0.3020	1.0686	1.1862	0.104*	
C34	0.2418 (3)	1.0812 (3)	1.0608 (2)	0.0748 (9)	
H34	0.1551	1.0622	1.0677	0.090*	
C35	0.0722 (3)	0.9308 (2)	0.8010 (2)	0.0586 (7)	
C36	0.1106 (4)	0.8400 (3)	0.8278 (3)	0.0833 (10)	
H36	0.1668	0.8509	0.8783	0.100*	
C37	0.0645 (5)	0.7317 (3)	0.7786 (3)	0.1062 (14)	
H37	0.0906	0.6703	0.7965	0.127*	
C38	−0.0173 (5)	0.7151 (3)	0.7056 (3)	0.1021 (15)	
H38	−0.0472	0.6424	0.6734	0.123*	
C39	−0.0565 (4)	0.8034 (3)	0.6785 (3)	0.0960 (13)	
H39	−0.1131	0.7914	0.6280	0.115*	
C40	−0.0119 (4)	0.9115 (3)	0.7263 (2)	0.0767 (9)	
H40	−0.0392	0.9721	0.7078	0.092*	

### Atomic displacement parameters (Å^2^)

**Table:**

	*U*^11^	*U*^22^	*U*^33^	*U*^12^	*U*^13^	*U*^23^
Sn1	0.04155 (11)	0.04929 (11)	0.04692 (10)	0.00313 (8)	0.00315 (7)	0.00384 (8)
Sn2	0.05062 (12)	0.04580 (11)	0.04939 (11)	0.00212 (8)	−0.00149 (8)	0.00462 (8)
S1	0.0731 (5)	0.0534 (4)	0.0516 (4)	0.0025 (3)	0.0115 (3)	0.0066 (3)
S2	0.0790 (5)	0.0557 (4)	0.0538 (4)	−0.0181 (4)	0.0069 (4)	−0.0015 (3)
O1	0.0422 (10)	0.0448 (10)	0.0654 (11)	−0.0024 (8)	−0.0035 (9)	0.0034 (8)
O2	0.0453 (11)	0.0628 (13)	0.0823 (14)	0.0190 (9)	−0.0079 (10)	−0.0113 (10)
C1	0.0486 (16)	0.0477 (15)	0.0582 (16)	−0.0006 (12)	0.0023 (12)	0.0028 (12)
C2	0.0427 (14)	0.0410 (13)	0.0520 (14)	0.0033 (11)	−0.0068 (11)	0.0037 (11)
C3	0.0398 (14)	0.0417 (13)	0.0562 (15)	0.0013 (10)	−0.0028 (11)	0.0021 (11)
C4	0.0485 (15)	0.0536 (15)	0.0513 (14)	0.0056 (12)	−0.0014 (12)	0.0007 (12)
C5	0.0468 (15)	0.0491 (14)	0.0481 (14)	0.0067 (12)	0.0008 (11)	0.0035 (11)
C6	0.0567 (17)	0.0518 (16)	0.0659 (17)	0.0019 (13)	0.0031 (14)	0.0103 (13)
C7	0.081 (2)	0.0490 (17)	0.072 (2)	0.0083 (15)	−0.0087 (17)	−0.0047 (14)
C8	0.080 (2)	0.069 (2)	0.068 (2)	0.0217 (18)	0.0108 (17)	−0.0089 (16)
C9	0.070 (2)	0.081 (2)	0.082 (2)	0.0082 (18)	0.0277 (18)	−0.0009 (18)
C10	0.0588 (19)	0.0565 (17)	0.075 (2)	−0.0015 (14)	0.0154 (15)	−0.0033 (14)
C11	0.0533 (16)	0.0550 (16)	0.0534 (15)	0.0082 (13)	−0.0033 (12)	0.0052 (12)
C12	0.066 (2)	0.082 (2)	0.0578 (17)	0.0133 (16)	−0.0049 (15)	0.0072 (15)
C13	0.098 (3)	0.108 (3)	0.0561 (19)	0.011 (2)	−0.0100 (19)	0.0087 (19)
C14	0.116 (4)	0.127 (4)	0.076 (3)	0.040 (3)	−0.035 (2)	0.009 (2)
C15	0.101 (3)	0.167 (5)	0.097 (3)	0.076 (3)	−0.026 (3)	−0.005 (3)
C16	0.077 (2)	0.134 (3)	0.070 (2)	0.047 (2)	−0.0063 (19)	−0.001 (2)
C17	0.0416 (14)	0.0411 (13)	0.0598 (15)	0.0053 (11)	0.0068 (12)	0.0037 (11)
C18	0.0582 (19)	0.076 (2)	0.0694 (19)	0.0011 (15)	0.0096 (15)	0.0120 (16)
C19	0.066 (2)	0.088 (3)	0.106 (3)	−0.0014 (19)	0.033 (2)	0.022 (2)
C20	0.0483 (19)	0.062 (2)	0.129 (3)	−0.0027 (15)	0.007 (2)	0.002 (2)
C21	0.057 (2)	0.0603 (19)	0.097 (3)	0.0046 (15)	−0.0134 (18)	−0.0009 (17)
C22	0.0535 (17)	0.0537 (16)	0.0671 (18)	0.0019 (13)	0.0014 (14)	0.0055 (13)
C23	0.0578 (17)	0.0542 (16)	0.0509 (15)	0.0075 (13)	0.0031 (13)	0.0143 (12)
C24	0.063 (2)	0.079 (2)	0.0688 (19)	0.0119 (17)	0.0007 (16)	0.0176 (16)
C25	0.066 (2)	0.111 (3)	0.093 (3)	0.026 (2)	0.0131 (19)	0.038 (2)
C26	0.100 (3)	0.100 (3)	0.106 (3)	0.048 (3)	0.037 (3)	0.030 (3)
C27	0.125 (4)	0.081 (3)	0.098 (3)	0.030 (3)	0.020 (3)	−0.010 (2)
C28	0.083 (2)	0.074 (2)	0.078 (2)	0.0142 (19)	−0.0018 (18)	−0.0088 (18)
C29	0.0599 (18)	0.0490 (15)	0.0561 (16)	0.0098 (13)	−0.0048 (13)	0.0054 (12)
C30	0.0617 (19)	0.0603 (17)	0.0657 (18)	0.0093 (14)	−0.0032 (15)	0.0055 (14)
C31	0.069 (2)	0.070 (2)	0.088 (2)	0.0108 (17)	−0.0189 (19)	0.0005 (18)
C32	0.095 (3)	0.072 (2)	0.077 (2)	0.016 (2)	−0.034 (2)	0.0019 (18)
C33	0.110 (3)	0.085 (2)	0.064 (2)	0.005 (2)	−0.012 (2)	0.0192 (18)
C34	0.075 (2)	0.083 (2)	0.066 (2)	−0.0013 (18)	−0.0056 (17)	0.0210 (17)
C35	0.0666 (19)	0.0454 (15)	0.0589 (16)	−0.0021 (13)	0.0115 (14)	0.0070 (12)
C36	0.109 (3)	0.0557 (19)	0.084 (2)	0.0129 (19)	0.014 (2)	0.0147 (17)
C37	0.154 (4)	0.055 (2)	0.108 (3)	0.011 (2)	0.031 (3)	0.018 (2)
C38	0.139 (4)	0.055 (2)	0.091 (3)	−0.030 (2)	0.041 (3)	−0.007 (2)
C39	0.113 (3)	0.073 (3)	0.080 (2)	−0.031 (2)	0.001 (2)	−0.0059 (19)
C40	0.092 (3)	0.0554 (18)	0.073 (2)	−0.0120 (17)	−0.0061 (18)	0.0047 (15)

### Geometric parameters (Å, °)

**Table:**

Sn1—C11	2.130 (3)	C17—C18	1.376 (4)
Sn1—C17	2.137 (3)	C17—C22	1.384 (4)
Sn1—C5	2.144 (2)	C18—C19	1.390 (5)
Sn1—S1	2.4159 (8)	C18—H18	0.9300
Sn2—C35	2.129 (3)	C19—C20	1.367 (5)
Sn2—C29	2.130 (3)	C19—H19	0.9300
Sn2—C23	2.133 (3)	C20—C21	1.365 (5)
Sn2—S2	2.4086 (8)	C20—H20	0.9300
S1—C1	1.818 (3)	C21—C22	1.381 (4)
S2—C4	1.832 (3)	C21—H21	0.9300
O1—C2	1.420 (3)	C22—H22	0.9300
O1—H1	0.8200	C23—C28	1.373 (4)
O2—C3	1.429 (3)	C23—C24	1.375 (4)
O2—H2	0.8200	C24—C25	1.382 (5)
C1—C2	1.518 (4)	C24—H24	0.9300
C1—H1A	0.9700	C25—C26	1.341 (6)
C1—H1B	0.9700	C25—H25	0.9300
C2—C3	1.525 (3)	C26—C27	1.362 (6)
C2—H2A	0.9800	C26—H26	0.9300
C3—C4	1.510 (4)	C27—C28	1.382 (5)
C3—H3	0.9800	C27—H27	0.9300
C4—H4A	0.9700	C28—H28	0.9300
C4—H4B	0.9700	C29—C30	1.380 (4)
C5—C10	1.379 (4)	C29—C34	1.386 (4)
C5—C6	1.385 (4)	C30—C31	1.385 (4)
C6—C7	1.382 (4)	C30—H30	0.9300
C6—H6	0.9300	C31—C32	1.350 (5)
C7—C8	1.363 (5)	C31—H31	0.9300
C7—H7	0.9300	C32—C33	1.358 (5)
C8—C9	1.357 (5)	C32—H32	0.9300
C8—H8	0.9300	C33—C34	1.391 (5)
C9—C10	1.378 (4)	C33—H33	0.9300
C9—H9	0.9300	C34—H34	0.9300
C10—H10	0.9300	C35—C40	1.376 (4)
C11—C16	1.377 (4)	C35—C36	1.378 (4)
C11—C12	1.381 (4)	C36—C37	1.393 (5)
C12—C13	1.384 (5)	C36—H36	0.9300
C12—H12	0.9300	C37—C38	1.347 (6)
C13—C14	1.351 (6)	C37—H37	0.9300
C13—H13	0.9300	C38—C39	1.356 (6)
C14—C15	1.352 (6)	C38—H38	0.9300
C14—H14	0.9300	C39—C40	1.382 (4)
C15—C16	1.377 (5)	C39—H39	0.9300
C15—H15	0.9300	C40—H40	0.9300
C16—H16	0.9300		
			
C11—Sn1—C17	114.46 (11)	C11—C16—H16	119.8
C11—Sn1—C5	107.59 (10)	C15—C16—H16	119.8
C17—Sn1—C5	104.55 (10)	C18—C17—C22	118.1 (3)
C11—Sn1—S1	108.60 (8)	C18—C17—Sn1	122.1 (2)
C17—Sn1—S1	118.70 (7)	C22—C17—Sn1	119.7 (2)
C5—Sn1—S1	101.47 (7)	C17—C18—C19	120.7 (3)
C35—Sn2—C29	113.96 (11)	C17—C18—H18	119.7
C35—Sn2—C23	113.26 (11)	C19—C18—H18	119.7
C29—Sn2—C23	109.18 (10)	C20—C19—C18	120.1 (3)
C35—Sn2—S2	107.68 (8)	C20—C19—H19	119.9
C29—Sn2—S2	105.19 (8)	C18—C19—H19	119.9
C23—Sn2—S2	107.00 (7)	C21—C20—C19	119.9 (3)
C1—S1—Sn1	101.39 (9)	C21—C20—H20	120.0
C4—S2—Sn2	106.59 (10)	C19—C20—H20	120.0
C2—O1—H1	109.5	C20—C21—C22	120.0 (3)
C3—O2—H2	109.5	C20—C21—H21	120.0
C2—C1—S1	112.91 (18)	C22—C21—H21	120.0
C2—C1—H1A	109.0	C21—C22—C17	121.2 (3)
S1—C1—H1A	109.0	C21—C22—H22	119.4
C2—C1—H1B	109.0	C17—C22—H22	119.4
S1—C1—H1B	109.0	C28—C23—C24	118.0 (3)
H1A—C1—H1B	107.8	C28—C23—Sn2	120.2 (2)
O1—C2—C1	108.2 (2)	C24—C23—Sn2	121.8 (2)
O1—C2—C3	111.3 (2)	C23—C24—C25	121.2 (3)
C1—C2—C3	111.7 (2)	C23—C24—H24	119.4
O1—C2—H2A	108.5	C25—C24—H24	119.4
C1—C2—H2A	108.5	C26—C25—C24	120.0 (4)
C3—C2—H2A	108.5	C26—C25—H25	120.0
O2—C3—C4	110.3 (2)	C24—C25—H25	120.0
O2—C3—C2	106.32 (19)	C25—C26—C27	120.2 (4)
C4—C3—C2	113.8 (2)	C25—C26—H26	119.9
O2—C3—H3	108.7	C27—C26—H26	119.9
C4—C3—H3	108.7	C26—C27—C28	120.3 (4)
C2—C3—H3	108.7	C26—C27—H27	119.8
C3—C4—S2	109.57 (18)	C28—C27—H27	119.8
C3—C4—H4A	109.8	C23—C28—C27	120.4 (4)
S2—C4—H4A	109.8	C23—C28—H28	119.8
C3—C4—H4B	109.8	C27—C28—H28	119.8
S2—C4—H4B	109.8	C30—C29—C34	118.3 (3)
H4A—C4—H4B	108.2	C30—C29—Sn2	121.2 (2)
C10—C5—C6	117.3 (2)	C34—C29—Sn2	120.5 (2)
C10—C5—Sn1	121.06 (19)	C29—C30—C31	120.6 (3)
C6—C5—Sn1	121.6 (2)	C29—C30—H30	119.7
C7—C6—C5	121.1 (3)	C31—C30—H30	119.7
C7—C6—H6	119.5	C32—C31—C30	120.0 (4)
C5—C6—H6	119.5	C32—C31—H31	120.0
C8—C7—C6	120.1 (3)	C30—C31—H31	120.0
C8—C7—H7	119.9	C31—C32—C33	121.1 (3)
C6—C7—H7	119.9	C31—C32—H32	119.4
C9—C8—C7	119.7 (3)	C33—C32—H32	119.4
C9—C8—H8	120.1	C32—C33—C34	119.6 (4)
C7—C8—H8	120.1	C32—C33—H33	120.2
C8—C9—C10	120.4 (3)	C34—C33—H33	120.2
C8—C9—H9	119.8	C29—C34—C33	120.4 (3)
C10—C9—H9	119.8	C29—C34—H34	119.8
C9—C10—C5	121.3 (3)	C33—C34—H34	119.8
C9—C10—H10	119.4	C40—C35—C36	118.7 (3)
C5—C10—H10	119.4	C40—C35—Sn2	120.7 (2)
C16—C11—C12	117.8 (3)	C36—C35—Sn2	120.5 (3)
C16—C11—Sn1	118.1 (2)	C35—C36—C37	119.6 (4)
C12—C11—Sn1	124.1 (2)	C35—C36—H36	120.2
C11—C12—C13	120.7 (3)	C37—C36—H36	120.2
C11—C12—H12	119.7	C38—C37—C36	120.6 (4)
C13—C12—H12	119.7	C38—C37—H37	119.7
C14—C13—C12	120.4 (4)	C36—C37—H37	119.7
C14—C13—H13	119.8	C37—C38—C39	120.6 (4)
C12—C13—H13	119.8	C37—C38—H38	119.7
C13—C14—C15	119.6 (4)	C39—C38—H38	119.7
C13—C14—H14	120.2	C38—C39—C40	119.6 (4)
C15—C14—H14	120.2	C38—C39—H39	120.2
C14—C15—C16	121.1 (4)	C40—C39—H39	120.2
C14—C15—H15	119.5	C35—C40—C39	120.9 (4)
C16—C15—H15	119.5	C35—C40—H40	119.6
C11—C16—C15	120.5 (4)	C39—C40—H40	119.6
			
C11—Sn1—S1—C1	−39.43 (12)	C22—C17—C18—C19	0.4 (5)
C17—Sn1—S1—C1	93.60 (12)	Sn1—C17—C18—C19	−175.9 (3)
C5—Sn1—S1—C1	−152.61 (12)	C17—C18—C19—C20	−0.9 (5)
C35—Sn2—S2—C4	−6.64 (13)	C18—C19—C20—C21	0.3 (6)
C29—Sn2—S2—C4	115.25 (12)	C19—C20—C21—C22	0.7 (5)
C23—Sn2—S2—C4	−128.71 (12)	C20—C21—C22—C17	−1.2 (5)
Sn1—S1—C1—C2	−73.4 (2)	C18—C17—C22—C21	0.6 (4)
S1—C1—C2—O1	65.3 (2)	Sn1—C17—C22—C21	177.0 (2)
S1—C1—C2—C3	−171.81 (18)	C35—Sn2—C23—C28	159.8 (3)
O1—C2—C3—O2	−70.6 (3)	C29—Sn2—C23—C28	31.7 (3)
C1—C2—C3—O2	168.3 (2)	S2—Sn2—C23—C28	−81.7 (3)
O1—C2—C3—C4	51.1 (3)	C35—Sn2—C23—C24	−21.5 (3)
C1—C2—C3—C4	−70.0 (3)	C29—Sn2—C23—C24	−149.7 (2)
O2—C3—C4—S2	−57.5 (2)	S2—Sn2—C23—C24	97.0 (2)
C2—C3—C4—S2	−176.88 (18)	C28—C23—C24—C25	0.2 (5)
Sn2—S2—C4—C3	121.26 (17)	Sn2—C23—C24—C25	−178.5 (3)
C11—Sn1—C5—C10	−83.1 (3)	C23—C24—C25—C26	−0.6 (6)
C17—Sn1—C5—C10	154.8 (2)	C24—C25—C26—C27	0.8 (6)
S1—Sn1—C5—C10	30.8 (2)	C25—C26—C27—C28	−0.7 (7)
C11—Sn1—C5—C6	94.9 (2)	C24—C23—C28—C27	−0.1 (5)
C17—Sn1—C5—C6	−27.2 (2)	Sn2—C23—C28—C27	178.6 (3)
S1—Sn1—C5—C6	−151.2 (2)	C26—C27—C28—C23	0.3 (7)
C10—C5—C6—C7	0.8 (4)	C35—Sn2—C29—C30	109.8 (2)
Sn1—C5—C6—C7	−177.2 (2)	C23—Sn2—C29—C30	−122.4 (2)
C5—C6—C7—C8	−0.4 (5)	S2—Sn2—C29—C30	−7.9 (2)
C6—C7—C8—C9	−0.3 (5)	C35—Sn2—C29—C34	−70.8 (3)
C7—C8—C9—C10	0.5 (6)	C23—Sn2—C29—C34	57.0 (3)
C8—C9—C10—C5	0.0 (6)	S2—Sn2—C29—C34	171.5 (2)
C6—C5—C10—C9	−0.6 (5)	C34—C29—C30—C31	1.1 (4)
Sn1—C5—C10—C9	177.4 (3)	Sn2—C29—C30—C31	−179.5 (2)
C17—Sn1—C11—C16	136.9 (3)	C29—C30—C31—C32	0.1 (5)
C5—Sn1—C11—C16	21.2 (3)	C30—C31—C32—C33	−1.1 (5)
S1—Sn1—C11—C16	−87.9 (3)	C31—C32—C33—C34	0.8 (6)
C17—Sn1—C11—C12	−42.2 (3)	C30—C29—C34—C33	−1.4 (5)
C5—Sn1—C11—C12	−157.9 (3)	Sn2—C29—C34—C33	179.2 (3)
S1—Sn1—C11—C12	93.0 (3)	C32—C33—C34—C29	0.5 (6)
C16—C11—C12—C13	−1.9 (5)	C29—Sn2—C35—C40	−177.6 (2)
Sn1—C11—C12—C13	177.2 (3)	C23—Sn2—C35—C40	56.8 (3)
C11—C12—C13—C14	1.6 (6)	S2—Sn2—C35—C40	−61.3 (3)
C12—C13—C14—C15	−0.5 (7)	C29—Sn2—C35—C36	−0.6 (3)
C13—C14—C15—C16	−0.3 (8)	C23—Sn2—C35—C36	−126.2 (3)
C12—C11—C16—C15	1.1 (6)	S2—Sn2—C35—C36	115.7 (2)
Sn1—C11—C16—C15	−178.0 (4)	C40—C35—C36—C37	0.5 (5)
C14—C15—C16—C11	0.0 (8)	Sn2—C35—C36—C37	−176.5 (3)
C11—Sn1—C17—C18	8.9 (3)	C35—C36—C37—C38	−0.3 (6)
C5—Sn1—C17—C18	126.3 (2)	C36—C37—C38—C39	0.0 (7)
S1—Sn1—C17—C18	−121.6 (2)	C37—C38—C39—C40	0.0 (6)
C11—Sn1—C17—C22	−167.4 (2)	C36—C35—C40—C39	−0.5 (5)
C5—Sn1—C17—C22	−49.9 (2)	Sn2—C35—C40—C39	176.5 (3)
S1—Sn1—C17—C22	62.2 (2)	C38—C39—C40—C35	0.3 (6)

### Hydrogen-bond geometry (Å, °)

**Table:**

*D*—H···*A*	*D*—H	H···*A*	*D*···*A*	*D*—H···*A*
O1—H1···O2^i^	0.82	1.95	2.745 (3)	163
O2—H2···C22^i^	0.82	2.80	3.493 (3)	143

Symmetry codes: (i) −*x*, −*y*+2, −*z*+1.
